# Mutations in *CECR1* associated with a neutrophil signature in peripheral blood

**DOI:** 10.1186/1546-0096-12-44

**Published:** 2014-09-24

**Authors:** Alexandre Belot, Evangeline Wassmer, Marinka Twilt, Jean-Christophe Lega, Leo AH Zeef, Anthony Oojageer, Paul R Kasher, Anne-Laure Mathieu, Christophe Malcus, Julie Demaret, Nicole Fabien, Sophie Collardeau-Frachon, Laura Mechtouff, Laurent Derex, Thierry Walzer, Gillian I Rice, Isabelle Durieu, Yanick J Crow

**Affiliations:** Hospices Civils de Lyon, et Université de Lyon, Lyon, France; CIRI, International Center for Infectiology Research, Inserm, U1111, Ecole Normale Supérieure de Lyon, Université Lyon 1, CNRS, UMR5308 Lyon, France; Centre de Référence des maladies Rénales Rares, Service de Néphrologie, Rhumatologie & Dermatologie Pédiatriques, Lyon, France; Department of Paediatric Neurology, Birmingham Children’s Hospital, Birmingham, UK; Department of Adolescent and Pediatric Rheumatology, Birmingham Children’s Hospital, Birmingham, UK; Department of Pediatrics, Division of rheumatology, Aarhus University Hospital, Aarhus, Denmark; Service de Médecine Interne et Vasculaire, Centre Hospitalier Lyon Sud, Lyon, France; Faculty of Life Sciences, University of Manchester, Manchester, UK; Manchester Centre for Genomic Médecine Interne et Vasculaire of Human Development Faculty of Medical and Human Sciences, Manchester Academic Health Sciences Centre, University of Manchester, Manchester, UK; Laboratoire d’Immunologie, Hôpital E. Herriot Lyon, Lyon, France; Laboratoire d’Immunologie, Centre Hospitalier Lyon Sud, Lyon, France; Centre de Pathologie Est, Groupement Hospitalier EST, Lyon, France; Service de Neurologie Vasculaire, Hôpital Pierre Wertheimer, Lyon, France; INSERM UMR 1163, Laboratory of Neurogenetics and Neuroinflammation, Paris Descartes – Sorbonne Paris Cité University, Institut Imagine, Hôpital Necker, Paris, France; Paris Descartes University, Paris, France; Laboratory of Neurogenetics and Neuroinflammation, Institut Imagine, 3rd Floor, Room 309, 24 boulevard du Montparnasse, 75015 Paris, France

**Keywords:** Adenosine deaminase, ADA2, CECR1, Neutrophil signature, Type I interferon, Aicardi-Goutières syndrome, SAMHD1

## Abstract

**Background:**

A reduction of ADA2 activity due to autosomal recessive loss of function mutations in *CECR1* results in a newly described vasculopathic phenotype reminiscent of polyarteritis nodosa, with manifestations ranging from fatal systemic vasculitis with multiple strokes in children to limited cutaneous disease in middle-aged individuals. Evidence indicates that ADA2 is essential for the endothelial integrity of small vessels. However, *CECR1* is not expressed, nor is the ADA2 protein detectable, in cultured human endothelial cells, thus implicating additional cell types or circulating factors in disease pathogenesis.

**Methods:**

Considering the phenotypic overlap of ADA2 deficiency with the type I interferonopathy Aicardi-Goutières syndrome due to mutations in *SAMHD1*, we looked for the presence of an interferon signature in the peripheral blood of two newly ascertained ADA2-deficient patients.

**Results:**

We identified biallelic *CECR1* mutations in two patients consistent with ADA2 deficiency. Both patients demonstrated an upregulation of interferon stimulated gene transcripts in peripheral blood. More strikingly however, genome-wide analysis revealed a marked overexpression of neutrophil-derived genes, suggesting that the vasculitis seen in ADA2 deficiency may be an indirect effect resulting from chronic and marked activity of neutrophils.

**Conclusions:**

We hypothesise that ADA2 may act as a regulator of neutrophil activation, and that a reduction of ADA2 activity results in significant endothelial damage via a neutrophil-driven process.

**Electronic supplementary material:**

The online version of this article (doi:10.1186/1546-0096-12-44) contains supplementary material, which is available to authorized users.

## Background

Autosomal recessive loss of function mutations in *CECR1*, encoding adenosine deaminase 2 (ADA2), were recently described to cause a variable phenotype encompassing recurrent fevers, livedo reticularis, Raynaud’s phenomenon and digital necrosis, vascular aneurysms and stenoses, intestinal strictures, hepatosplenomegaly and hypogammaglobulinemia [1, 2]. Biopsies of affected tissues revealed an inflammatory vasculopathy showing overlap with polyarteritis nodosa as a major pathological feature, and signs of compromised endothelial integrity and endothelial cellular activation. The precise mechanism leading to disease remained undefined, but was suggested to relate to a function of ADA2 as an immune system molecule and/or as a growth factor.

Here we report two further patients with biallelic mutations in *CECR1*. In view of a clinical overlap with the Mendelian type I interferonopathy disease Aicardi-Goutières syndrome (AGS) due to mutations in *SAMHD1*, we searched for an interferon signature in peripheral blood [3, 4]. Both patients demonstrated an upregulation of interferon stimulated gene transcripts. However, more strikingly, we observed a marked overexpression of neutrophil-related genes on genome-wide microarray analysis, and circulating neutrophils demonstrated increased expression of myeloperoxidase compared to controls. Neutrophil counts were normal. These findings suggests that ADA2 deficiency may drive unregulated neutrophil activation, and thus play a role in the activation of endothelial cells and the pathogenesis of the vasculitis of this devastating disease.

## Methods

For Sanger sequencing, primers were designed to amplify the coding exons of *CECR1* (see Additional file 1: Table S1). Purified PCR amplification products were sequenced using BigDye™ terminator chemistry and an ABI PRISM 3730 xL Genetic Analyzer (96-capillary system). Mutation description was based on the reference cDNA sequence NM_017424.2, with nucleotide numbering beginning from the first A in the initiating ATG codon.

Methods for the assessment of the expression of a panel of interferon stimulated genes (ISGs) have been described previously [4]. Briefly, total RNA was extracted from blood (peripheral blood mononuclear cells) using a PAXgene (PreAnalytix) RNA isolation kit. Quantitative reverse transcription polymerase chain reaction (qPCR) analysis was performed using the TaqMan Universal PCR Master Mix (Applied Biosystems), and cDNA derived from 40 ng total RNA. Using TaqMan probes for *IFI27* (Hs01086370_m1), *IFI44L* (Hs00199115_m1), *IFIT1* (Hs00356631_g1), *ISG15* (Hs00192713_m1), *RSAD2* (Hs01057264_m1), and *SIGLEC1* (Hs00988063_m1), the relative abundance of each target transcript was normalized to the expression level of *HPRT1* (Hs03929096_g1) and *18S* (Hs999999001_s1), and assessed with the Applied Biosystems StepOne Software v2.1 and DataAssist Software v.3.01. The median fold change of the six ISGs, when compared to the median of 29 healthy controls combined, was used to create an interferon score for each patient. RQ is equal to 2^-ΔΔCt^ i.e. the normalized fold change relative to a control. When a patient was assayed on more than one occasion, the data for repeat measurements were combined to calculate a mean value. The mean interferon score of the controls plus two standard deviations above the mean (+2 SD) was calculated. Scores above this value (>2.466) were designated as positive. Neutrophil-expressed genes were assessed as above using Taqman probes for *MMP8* (Hs01029057_m1), *CEACAM6* (Hs03645554_m1), *CRISP3* (Hs00195988_m1), *LTF* (Hs00914334_m1), *DEFA4* (Hs01056651_g1) and *LCN2* (Hs01008571_m1). Neutrophil-expressed gene expression in patients was normalized to expression in 4 healthy control samples.

RNA quality was checked using the RNA 6000 Nano Assay, and analyzed on an Agilent 2100 Bioanalyser (Agilent Technologies). RNA was quantified using a Nanodrop ultra-low-volume spectrophotometer (Nanodrop Technologies). Human Genome U133 Plus 2.0 Affymetrix GeneChips were run according to manufacturers instructions. RNA-Seq data was generated on a hiSeq 2500 using SENSE mRNA-seq library Prep kit (Lexogen).

For the microarray analysis, technical quality control and outlier analysis was performed with dChip (V2005) (http://www.hsph.harvard.edu/cli/complab/dchip/) [5], using the default settings. Background correction, quantile normalization, and gene expression analysis were performed using RMA in Bioconductor [6].

For the RNA-Seq analysis, quality assessment was performed with FastQC software (http://www.bioinformatics.babraham.ac.uk/projects/fastqc/). Mapping of reads to the human transcriptome was done with TopHat software [7] and transcript assembly and differential expression will be performed with Cuffdiff of Cufflinks [7]. Read counts were normalised with DESeq [8].

ADA2 activity in serum was assessed using a commercial kit (Diazyme Laboratories) based on the deamination of adenosine to inosine according to the manufacturer’s instructions.

The PerFix-no centrifuge assay Kit from Beckman Coulter (Hialeah, FL, USA) was used to measure myeloperoxidase (MPO) intracellular concentration. Staining of fresh whole blood was performed using Fluorescein IsoThioCyanate (FITC)-labeled anti-myeloperoxidase (clone CLB-MPO-1), Phycoerythrin (PE)-labeled anti-lactoferrin (clone CLB13.17), PE-Texas Red (ECD)-labeled anti-CD62L (clone DREG56), PE-Cyanine7 (PC7)-labeled anti-CD10 (clone ALB1), APC-Alexa fluor 750 (AA750)-labeled anti-CD11b (clone Bear1) and Pacific Blue (PB)-labeled anti-CD16 (clone 3G8). All reagents were purchased from Beckman Coulter. According to the manufacturer’s instructions, samples were first fixed with the Fixative Reagent and incubated for 15 min. Then, aliquots were simultaneously permeabilized and stained with fluorochrome-conjugated antibodies. After 35 min of incubation, samples were fixed using a solution containing formaldehyde. Cytometry analyses were performed on a NAVIOS flow cytometer using the NAVIOS software (Beckman Coulter).

The study was approved by a U.K. Multicentre Research Ethics Committee (reference number 04:MRE00/19) and the Comité de protection des personnes SUD-EST III (reference number 2013-011B).

### Case studies

#### Patient 1 (F785)

**Figure 1:**
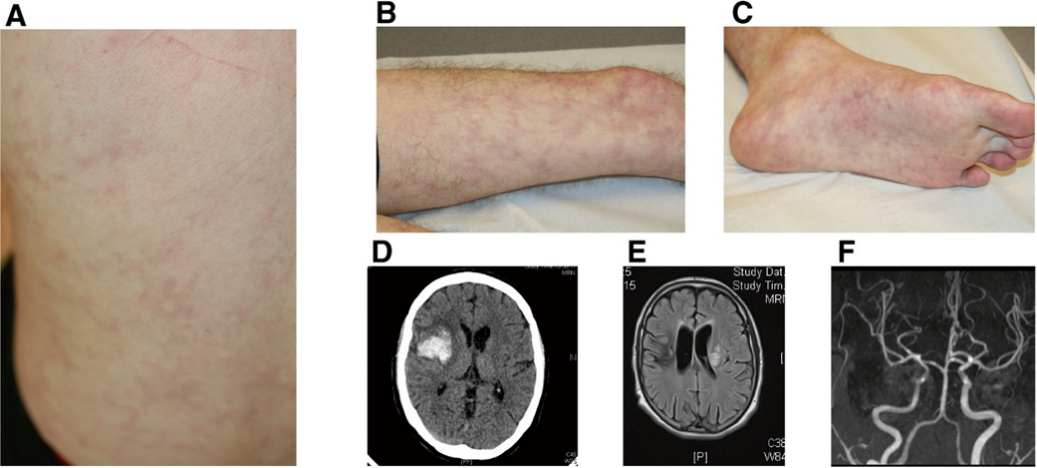
**Clinical and radiological features. A-C**. Generalized livedo of the back **(A)**, leg **(B)** and foot **(C)**. **D**. Right frontal insular haemorrhage. **E**. Left middle cerebral ischemic stroke. **F**. Left internal carotid artery stenosis.

This male was the last of five children born to non-consanguineous parents of European French ancestry. Of note, an older brother died at the age of 28 years having exhibited lifelong inflammatory features including livedo patterning of the legs, necrotic lesions of the limbs and multiple cerebral vascular accidents.Patient 1 presented soon after birth with recurrent episodes of unexplained fever, together with apthous ulcers of the mouth and joint pains. At the age of 11 years he developed marked livedo patterning of the arms and legs (Figure 1). At this time he experienced a transient neurological ischemic attack in the context of a febrile illness. Autoantibody levels (ANCA, ANA, anti-dsDNA) were negative. He demonstrated a significant IgG deficiency with a slight decrease in IgM and IgA titres. IgE levels were mildly elevated. Extensive investigations were otherwise non-contributory. At the age of 12 years he presented with mononeuritis multiplex (median nerve, external popliteal nerve), with accompanying indices of systemic inflammation (increased ESR and CRP). He was treated with oral steroids, but after an extension of his mononeuritis he subsequently received 6 pulses of monthly cyclophosphamide with partial improvement. IVIG replacement therapy was started at this time.

At the age of 15 years he experienced an acute abdominal crisis requiring surgery. This revealed stenosis of the ileo-caecum with evidence of a necrotizing vasculitis of the small and medium sized vessels evocative of polyarteritis nodosa (Additional file 1: Figure S1). Soon thereafter he suffered an acute cerebrovascular accident resulting in a left-sided hemiparesis. Treatment with methotrexate and azathioprine was successfully introduced; but both were stopped because of side effects. Steroids were maintained. He developed a further motor deficit of the right arm with dysarthria a year later, at which point ischemic lesions of the left thalamus were noted on cranial magnetic resonance imaging. The introduction of rituximab did not prevent the occurrence of a further stroke-like episode. Subsequently, he developed a hemorrhagic lesion of the spleen whist on anticoagulant therapy. At the age of 21 years he presented with an intracerebral hemorrhage. Cutaneous peri-malleolar ulcerative lesions were noted at the age of 23 years. He was growth retarded with a height of 150 cm (−−2 SDs below the mean).

#### Patient 2 (F750)

This male child was born to consanguineous parents of Pakistani ancestry with no family history of note. Between the ages of 3 weeks and 2 years he experienced recurrent episodes of dactylitis of both hands mainly involving the metacarpophalangeal joints. From the age of 2 years he developed fevers lasting 4–5 days, associated with a raised CRP and ESR. Erythema nodosum was noted from the age of 2.5 years on all 4 limbs, but particularly affecting the legs. Biopsy demonstrated septal panniculitis.

He complained of intermittent headaches from the age of 4 years. Against a background of normal development, at age 6 years he suffered an intracranial hemorrhage treated with evacuation, leaving him with a right hemiplegia and speech impairment. Following the acute event he continued to have intermittent fever and raised ESR/CRP. He was also noted to have unexplained hypertension, requiring anti-hypertensive medication, and a vasculitic rash. Three months later he suffered 2 further acute neurological events, leaving him with a left and right third nerve palsy with optic neuritis. Cranial magnetic resonance imaging demonstrated multiple white matter abnormalities with linear enhancement and a left frontal porencephalic cyst. Over time, some of these lesions resolved and new lesions appeared. Angiography revealed multiple microaneurysms within the sub-segmental branches of multiple vessels in the liver, kidneys and superior mesenteric artery consistent with a vasculitis-type pattern. At age 6 years, cerebrospinal fluid (CSF) cells, oligoclonal bands and alpha interferon activity were normal. CSF pterins demonstrated a normal tetrahydrobiopterin and dihydrobiopterin, but an increased neopterin of >4 times the upper limit of normal (297 nmol/l: control 7 – 65).

Considering an inflammatory disorder, he was treated with intravenous steroids to which he made a positive response, with an improvement in his cranial neuropathy, and a settling of his fever and inflammatory markers. He was then started on steroids, mycophenolate mofetil and, as he remained steroid dependent, a course of cyclophosphamide.

## Results

In patient 1, Sanger sequencing revealed compound heterozygosity for two variants (c.506G > A, p.Arg169Gln; c.578C > T, p.Pro193Leu) in *CECR1*. The Arg169Gln mutation has been reported previously in the compound heterozygous state in 3 families [2]. The Pro193Leu is novel but is considered likely pathogenic on the basis of conservation and prediction packages and its absence from publically available control sequence databases (Additional file 1: Figure S2, Additional file 1: Table S2). Each parent was heterozygous for one mutation.

Consistent with ADA2 deficiency, ADA2 activity was markedly decreased in serum (Additional file 1: Figure S3).

**Figure 2:**
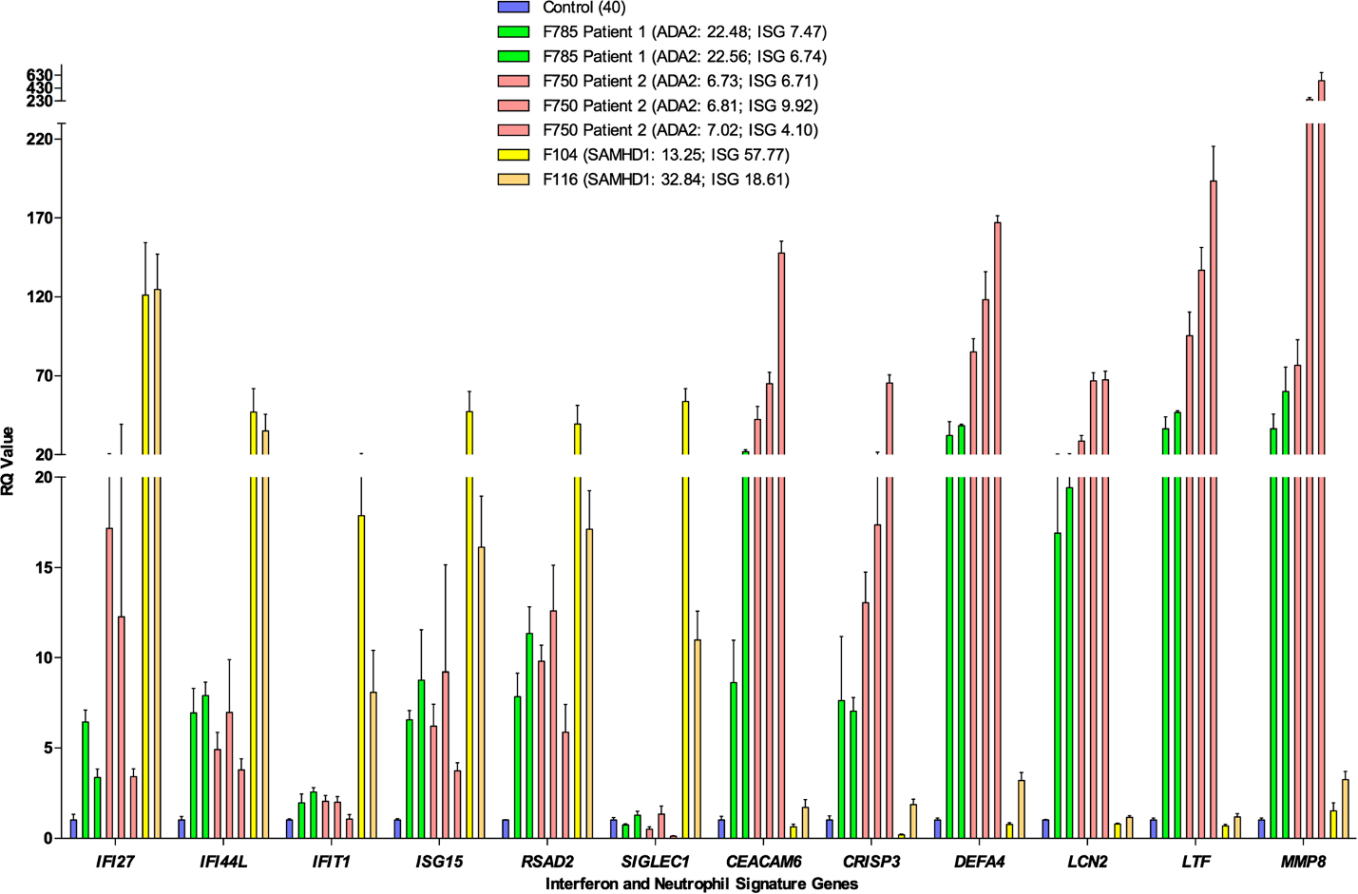
**Gene expression studies.** Quantitative reverse transcription PCR (qPCR) of a panel of six interferon stimulated genes (ISGs) and six neutrophil-expressed genes in whole blood measured in CECR1 mutation-positive probands, SAMHD1 mutation-positive probands and controls. Bar graph showing relative quantification (RQ) values for a panel of six interferon stimulated genes (ISGs) (IFI27, IFI44L, IFIT1, ISG15, RSAD2, SIGLEC1) and six neutrophil-expressed genes (CEACAM6, CRISP3, DEFA4, LCN2, LTF, MMP8) measured in whole blood in two ADA2 mutation positive patients, and two SAMHD1 mutation positive patients, compared to a healthy control. RQ is equal to 2-∆∆Ct, with -∆∆Ct ± standard deviations (i.e. the normalized fold change relative to a calibrator). Each value is derived from three technical replicates. Numbers in brackets refer to decimalized age at sampling, followed by interferon score calculated from the median fold change in relative quantification value for the panel of six ISGs. Colors denote individuals, with repeat samples (biological replicates) denoted by different bars of the same color.

**Figure 3:**
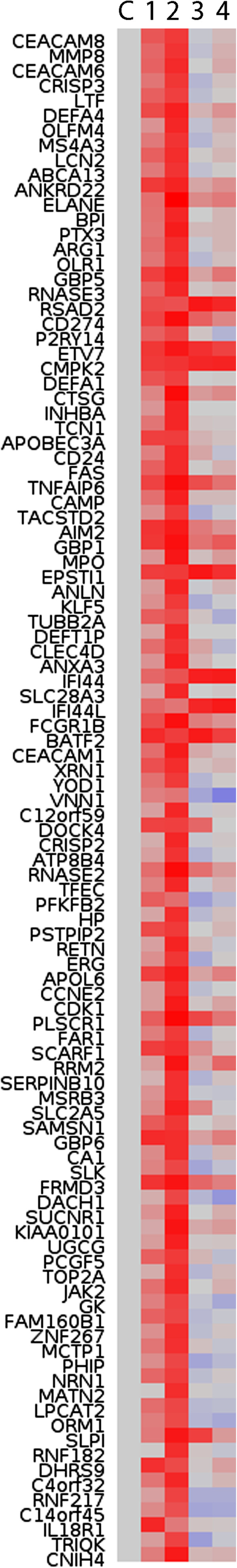
**Genome-wide gene expression.** Heatmap showing genome-wide expression array in patients with mutations in *CECR1* and RNA-seq analysis in patients with mutations in *SAMHD1*. Genes shown had a fold change greater than 5 (mean patients versus mean controls in the microarrays). Normalised expression levels for the microarray were expressed as fold change versus control samples in log scale. Similarly, for RNA-seq, normalised counts were expressed as fold change versus control samples in log scale. To generate the heatmap these log ratios (and control values of zero) were normalised between genes by setting the standard deviation of each gene to 1. Blue low expression, grey no change, red high expression. Samples shown: C = control; 1 = F785 patient 1 (ADA2); 2 = F750 patient 2 (ADA2); 3 = F104 (SAMHD1); 4 = F116 (SAMHD1).

We measured the expression of ISGs in peripheral blood (Figure 2). Interferon scores of 5.7 and 5.0 were recorded at ages 22.48 and 22.56 years respectively. The patient’s parents, tested on one occasion, did not demonstrate an interferon signature (interferon score of 0.6 in the father at age 66 years and 0.4 in the mother at 64 years). Genome-wide expression array showed a marked upregulation of neutrophil-expressed genes, and these findings were confirmed on qPCR of a selection of 6 of the most upregulated genes identified in the array data (Figures 2 and 3). In line with these findings, intracellular staining revealed an increased expression of MPO in peripheral blood mononuclear (PMN) cells compared to controls (Mean Fluorescence Intensity = 13.5; normal range [2.9-8]) (Additional file 1: Figure S4). Other markers, including lactoferrin, CD10, CD11b, CD16 and CD62L, were within normal range (data not shown).

In patient 2, Sanger sequencing, revealed a homozygous c.139G > C/p.Gly47Arg mutation in *CECR1*. This particular nucleotide variant has been reported in the compound heterozygous state in one Turkish patient [9], and results in the same amino acid substitution as a Georgian Jewish founder mutation (c.139G > A) described previously [1]. This variant has not been seen in the EVS control database, is highly conserved and is predicted to be damaging (Additional file 1: Figure S2, Additional file 1: Table S2). Both parents were heterozygous for the variant. Interferon scores of 5.1, 7.0 and 3.3 were recorded at 6.73, 6.81 and 7.02 decimalised years of age respectively. Parental samples were not available for testing. A genome-wide expression array demonstrated marked upregulation of neutrophil-expressed genes (Figure 3), and these findings were confirmed on qPCR of a selection of 6 of the most upregulated genes identified in the array data (Figure 2).

## Discussion

Autosomal recessive loss of function mutations in *CECR1*, leading to a reduction of ADA2 activity in patient sera, result in a vasculopathic phenotype reminiscent of polyarteritis nodosa showing highly variable age at onset, severity and organ involvement, even within families and among patients with the same mutations. Manifestations range from fatal systemic vasculitis with multiple strokes in children, to limited cutaneous disease in middle-aged individuals. A tendency to aneurysmal and stenosis formation in the brain, heart, kidney and gut vasculature results in a substantial risk of both infarction and haemorrhage. Inflammation was noticed in most patients suggesting that the underlying mechanism is driven by immune cells [1, 2].

The clinical features observed in our patients conform to the phenotype of ADA2 deficiency already reported, and include recurrent fevers, livedo reticularis, ulcerative skin lesions/erythema nodosum, hypogammagobulinaemia, gastrointestinal strictures with microaneuryms and intracerebral vascular disease. We identified compound heterozygous *CECR1* mutations in one patient, including a p.Arg169Gln substitution which has been previously seen in three families. Our second patient was homozygous for a c.139G > C/p.Gly47Arg mutation. This particular nucleotide variant has been reported in the compound heterozygous state in one Turkish patient, and results in the same amino acid substitution as a Georgian Jewish founder mutation, indicating the importance of the glycine at position 47 for enzyme activity (Additional file 1: Figure S5).

In 2009, we identified mutations in *SAMHD1* to cause a subtype of AGS [3], an inflammatory disease of the brain and skin associated with an upregulation of type I interferon signaling [4]. Clinically, SAMHD1-related AGS differs from other AGS subtypes in that many patients, more than 20 described in the literature so far - ranging in age from neonate to adult, demonstrate a cerebral vasculopathy which can manifest both as stenotic, leading to a moyamoya appearance, and aneurysmal disease, which carry a high risk of intracerebral infarction and haemorrhage respectively [10–13]. In our experience, patients with SAMHD1-related intracerebral vascular disease invariably demonstrate marked peripheral skin involvement, reminiscent of the cutaneous lesions described in ADA2 deficiency, with features of a leucocytoclastic vasculitis on biopsy.

SAMHD1 acts as a deoxynucleoside triphosphate triphosphohydrolase which regulates the intracellular pool of dNTPs, and hence the production of DNA through reverse transcription in the cytosol [14]. Patients with AGS consistently demonstrate an increased expression of ISGs, a so-called interferon signature, in peripheral blood [4]. These observations are important in identifying AGS as an inflammatory disorder associated with the induction of a type I interferon mediated innate immune response, likely driven by endogenously-derived nucleic acids. It is of note that patients with AGS consistently demonstrate an upregulation of pterins [15], an inflammatory marker, in the cerebrospinal fluid, a feature also observed in our patient 2.

Considering the overlap between the ADA2 and SAMHD1 clinical and radiological phenotypes, we looked for the presence of an interferon signature in the peripheral blood of the two ADA2-deficient patients that we ascertained. Both demonstrated an upregulation of ISG transcripts, which was present on all three occasions in patient 1, and on two occasions in patient 2 when assayed. More strikingly however, genome-wide analysis revealed a marked overexpression of neutrophil-expressed genes. This finding was confirmed on qPCR of a selection of 6 of the most upregulated genes identified in the array data. In a previous report, extensive studies of serum cytokines, and of cytokines produced by cultured peripheral-blood mononuclear cells, did not demonstrate any convincing differences between ADA2 deficient patients and controls [2]. Of note, an analysis of gene expression in peripheral blood was not reported. Here we show that ADA2 deficiency is associated with a marked upregulation of neutrophil expressed gene transcripts, which is distinct from the gene expression pattern seen in another monogenic inflammatory vasculitis due to mutations in *SAMHD1*.

## Conclusion

Previous reports indicate that ADA2 is essential for the endothelial integrity of small vessels. However, *CECR1* is not expressed, nor is the ADA2 protein detectable, in cultured human endothelial cells, thus implicating additional cell types, or circulating factors, in the disease pathogenesis [2]. ADA2 is a secreted molecule which converts adenosine to inosine and 2′-deoxyadenosine to 2′-deoxyinosine [16, 17]. Neutrophils express receptors for adenosine which can have profound effects on their function [18]. Our findings suggest that the vasculitis seen in ADA2 deficiency may be an indirect effect resulting from chronic and marked activity of neutrophils. As one possibility, MPO is a highly abundant neutrophil protein that has been recently reported as key disrupter of endothelial cell function [19]. ADA2 may act as a plasmatic regulator of PMN cell activation and prevent MPO expression and subsequent endothelial damage.

### Consent

Written informed consent was obtained from the patient and both parents (patient 1), and the family of patient 2 for the publication of this report and the accompanying images. The study was approved by the Leeds (East) Research Ethics Committee (reference number 10/H1307/132) and the Comité de protection des personnes SUD-EST III (reference number 2013-011B).

## Electronic supplementary material

Additional file 1: **Primers to amplify the coding exons of** ***CECR1.*** (PDF 2 MB)
