# Supplementary material for: Mutations in CECR1 associated with a neutrophil signature in peripheral blood
Source: Pediatr Rheumatol Online J. 2014 Sep 24;12:44. doi: 10.1186/1546-0096-12-44 (PMC4181355; doi:10.1186/1546-0096-12-44)
Supplement: Supplementary file 1 — Additional file 1: Primers to amplify the coding exons of CECR1. (PDF 2 MB) [file 12969_2014_2167_MOESM1_ESM.pdf]

Supplementary Information

Mutations in *CECR1* associated with a neutrophil signature in peripheral blood

Alexandre Belot[1,2,3], Evangeline Wassmer[4,] Marinka Twilt[5,6], Jean-Christophe Lega[1,7], Leo AH Zeef[8], Anthony Oojageer[9], Paul R Kasher[9], Anne-Laure Mathieu[2], Christophe Malcus[1,10],Julie Demaret[1,10], Nicole Fabien[1,11], Sophie Collardeau-Frachon[1,12], Laura Mechtouff[1,13], Laurent Dereix[1,13], Thierry Walzer[2], Gillian I Rice[9], Isabelle Durieu[1,7], Yanick J Crow[9,14,15]

[1]Hospices Civils de Lyon, et Université Lyon 1, France
[2]CIRI, International Center for Infectiology Research, Inserm, U1111, Ecole Normale Supérieure de Lyon, Université Lyon 1, CNRS, UMR5308, Lyon, France
[3]Centre de référence des maladies rénales rares, Service de Néphrologie, Rhumatologie & Dermatologie pédiatriques, Lyon, France
[4]Department of Paediatric Neurology, Birmingham Children's Hospital, Birmingham, UK
[5]Department of Adolescent and Pediatric Rheumatology, Birmingham Children’s Hospital, Birmingham, UK
[6]Division of rheumatology, department of pediatrics, Aarhus University Hospital, Aarhus, Denmark
[7]Service de Médecine Interne, Centre Hospitalier Lyon Sud, Lyon, France
[8]University of Manchester, Faculty of Life Sciences, Manchester, UK
[9]Manchester Centre for Genomic Medicine, Institute of Human Development Faculty of Medical and Human Sciences, Manchester Academic Health Sciences Centre, University of Manchester, UK
[10]Laboratoire d'Immunologie, Hôpital E. Herriot Lyon, France
[11]Centre Hospitalier Lyon Sud, Laboratoire d’Immunologie, Lyon, France
[12]Service d’Anatomie Pathologique, Groupe Hospitalier Est, Lyon, France
[13]Service de Neurologie Vasculaire, Hôpital Pierre Wertheimer, Lyon, France
[14]INSERM UMR 1163, Laboratory of Neurogenetics and Neuroinflammation, Paris Descartes – Sorbonne Paris Cité University, Institut Imagine, Hôpital Necker, Paris, France
[15]Paris Descartes University, Paris, France

Corresponding author and address:
Professor Y J Crow

Laboratory of Neurogenetics and Neuroinflammation
Institut Imagine
3rd Floor, Room 309
24 boulevard du Montparnasse
75015 Paris
Email: yanickcrow@mac.com

Phone: +33 1 42 75 44 51

Supplementary Table 1. Primers used to amplify *CECR1* exons (including intron/exon boundaries)

Exon	Primer name	Sequence
Exon 1	CECR1_1F	CAGGGCGAGAAAAGGAAGAT
	CECR1_1R	CCCCAAGATGAGTCCCTCTA
Exon 2	CECR1_2F	ACTTCACCCCCTCCTTTGTC
	CECR1_2R	TCTATAGGTTTGTACCAAGGGAGA
Exon 3	CECR1_3F	ATGCAAGGTGGGTAGCAGTC
	CECR1_3R	TGGTCAATTCATGAGCATTCA
Exon 5	CECR1_6F	GAGTTGACTGGGAAGGAGGT
	CECR1_6R	CACCGCACTTACCTAGGACTG
Exon 6	CECR1_7F	AAACGGGACCTGCACACTT
	CECR1_7R	CTCCCTGAATGCCATCCTTA
Exon 7	CECR1_8F	GGCTGTAGTGCATTGGTGTG
	CECR1_8R	ATGGGGCTGTTGTCAGGAT
Exon 8	CECR1_9F	GGGGGCTGTTTATTTGAGGA
	CECR1_9R	AGGAGAGTGGAGGGATGTAGG
Exon 9	CECR1_10F	GGAGCATAAGGACTGGCTCT
	CECR1_10R	AGAAGGAACCCACAGGAACC
Exon 10a	CECR1_11F	TGCTCTGCAAGGCTCTAATG
	CECR1_11R	TGGAGCTGATTCAAGAACGA

Transcript NM_017424.2

Supplementary Table 2: Results of SIFT, PolyPhen and Align GVGD analysess of the effects of the amino acid substitutions observed in *CECR1* mutation-positive patients

cDNA	Genomic DNA Chr22 (GRch37):	Protein	SIFT	PolyPhen	Align GVGD
c.139G>C	g.17690429C>G	p.Gly47Arg	Deleterious score 0	Probably damaging score 1	Class C0 unlikely to be damaging
c.506G>A	g.17687997C>T	p.Arg169Gln	Deleterious score 0	Probably damaging score 1	Class C0 unlikely to be damaging
c.578C>T	g.17684628G>A	p.Pro193Leu	Deleterious score 0	Probably damaging score 1	Class C0 unlikely to be damaging

SIFT: http://sift.jcvi.org/www/SIFT_enst_submit.html

PolyPhen 2: http://genetics.bwh.harvard.edu/pph2/

Align GVGD: http://agvgd.iarc.fr/index.php

SIFT score ranges from 0 to 1. The amino acid change is predicted to be damaging if the score is ≤ 0.05, and tolerated if the score is >0.05

PolyPhen 2 score ranges from 0 to 1, with the threshold for probably damaging at 0.85

Align GVGD classes mutations into a spectrum (C0, C15, C25, C35, C45, C55, C65), with C65 the most likely to interfere with function and C0 the least likely

Supplementary Figure 1.

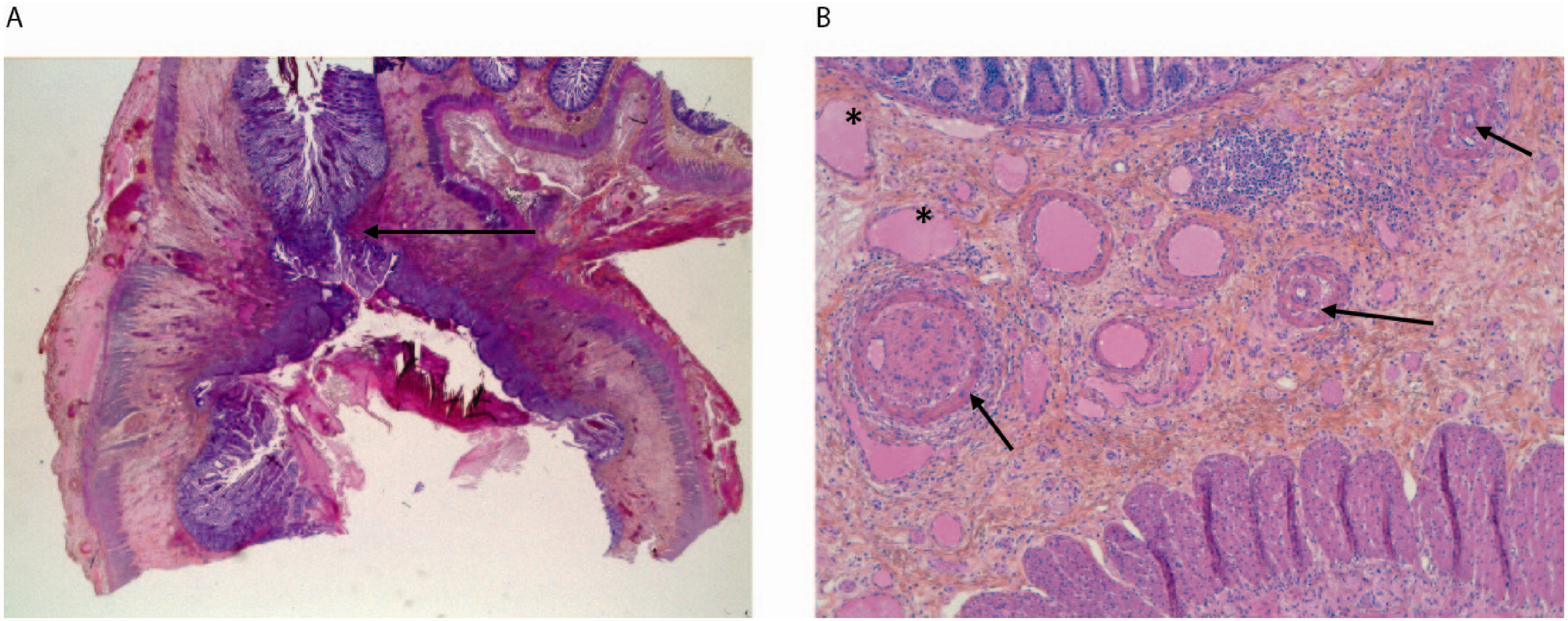

A. Small bowel vasculitis, ileal stenosis (black arrow), HES x10. B. Necrotizing vasculitis of gut mucosa with arterial obliterations (arrows) and normal veins (asterix), HESx50

Supplementary Figure 2.

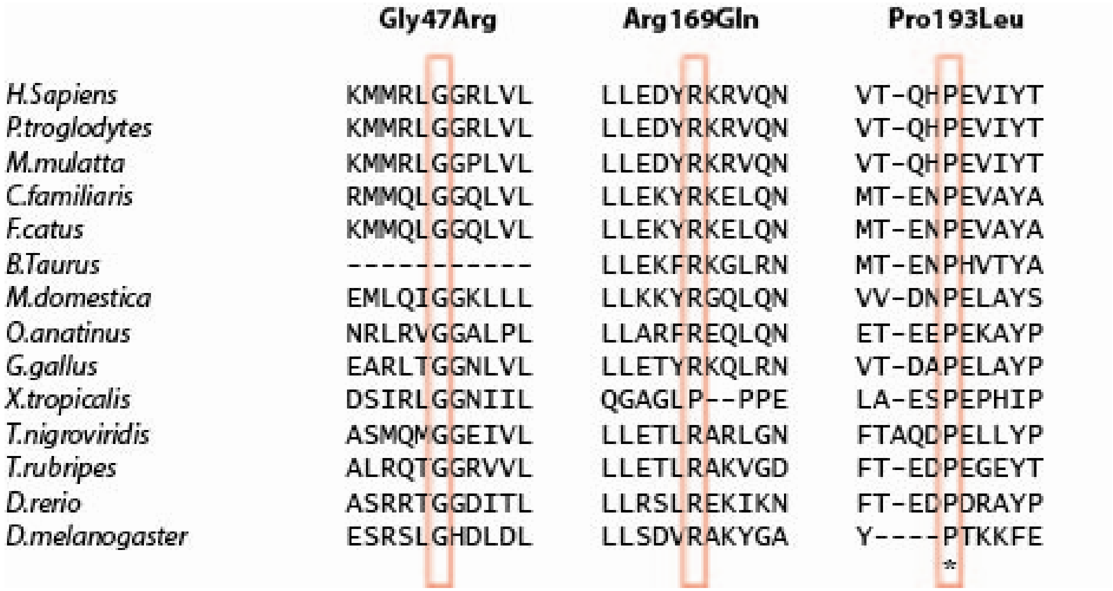

Figure x. CLUSTAL Omega alignment of ADA2 homologs. ADA2 homologs were identified on Ensembl and aligned using CLUSTAL Omega. Amino acids altered by CECR1 mutations are highlighted by a red box. *H.Sapiens*, *Homo sapiens* (ENSP00000382733); *P.troglodytes*, *Pan troglodytes* (ENSPTRG00000014036); *M.mulatta*, *Macaca mulatta* (ENSMMUG00000002529); *C.familiaris*, *Canis lupus familiaris* (ENSCAFG00000016114); *F.catus*, *Felis catus* (ENSFCAG00000012168); *B.Taurus*, *Bos Taurus* (ENSBTAG00000017313); *M.domestica*, *Monodelphis domestica* (ENSMODG00000018443); *O.anatinus*, *Ornithorhynchus anatinus* (ENSOANG00000002303); *G.gallus*, *Gallus gallus* (ENSGALG00000013031); *X.tropicalis*, *Xenopus tropicalis* (ENSXETG00000000874); *T.nigroviridis*, *Tetraodon nigroviridis* (ENSTNIG00000008799); *T.rubripes*, *Takifugu rubripes* (ENSTRUG00000011247); *D.rerio*, *Danio rerio* (ENSDARG00000051746); *D.melanogaster*, *Drosophila melanogaster* (FBgn0036752). Homology to the human ADA2 reference sequence (ENSP00000382733): human-*P.troglodytes*, 99%; human-*M.mulatta*, 90%; human-*C.familiaris*, 71%; human-*F.catus*, 72%; human-*B.Taurus*, 67%; human-*M.domestica*, 65%; human-*O.anatinus*, 49%; human-*G.gallus*, 56%; human-*X.tropicalis*, 43%; human-*T.nigroviridis*, 48%; human-*T.rubripes*, 50%; human-*D.rerio*, 50%; human-*D.melanogaster*, 37%.

Supplementary Figure 3.

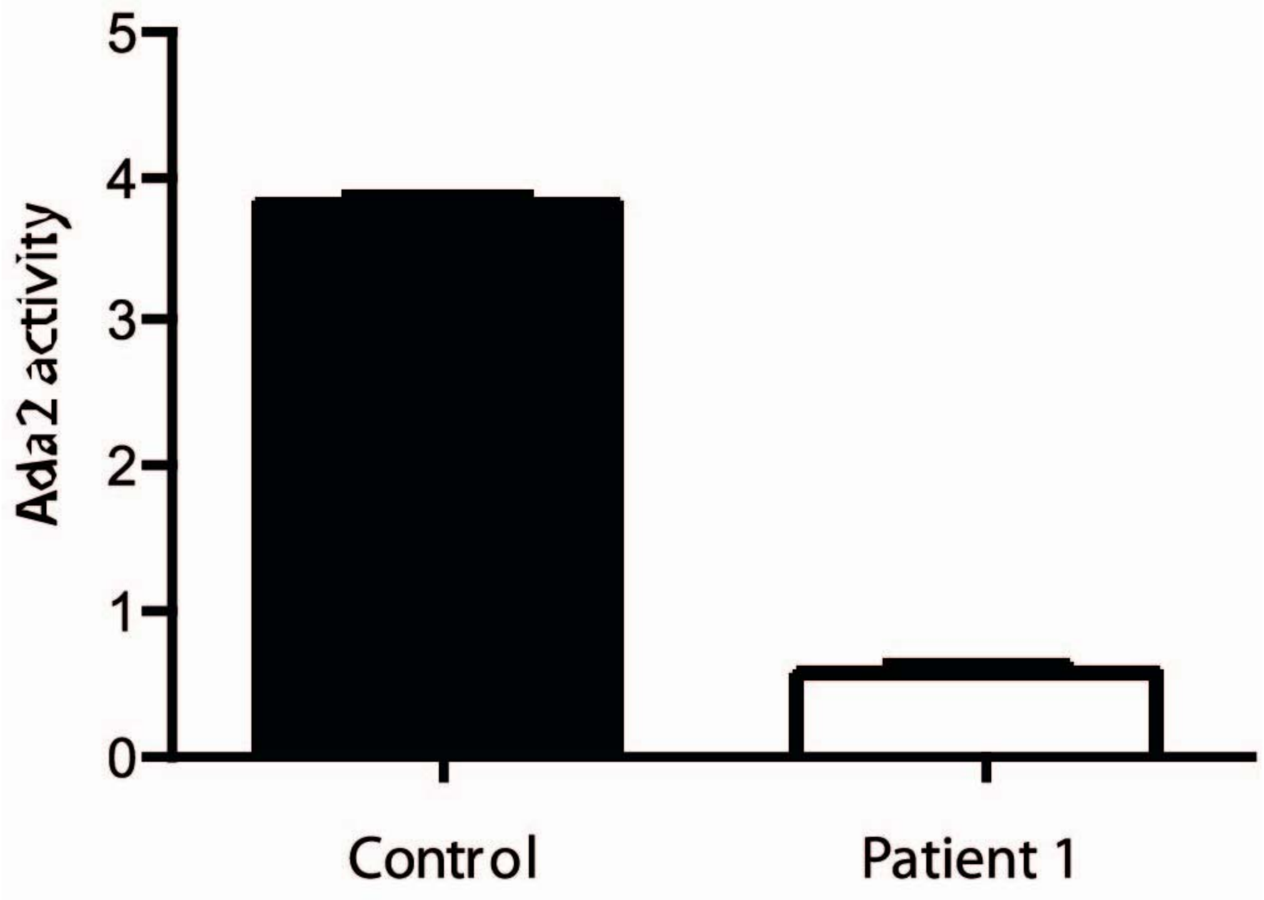

ADA2 activity: Pro193Leu/Arg169Gln compound heterozygous patient displays reduced activity of ADA2 in serum.

Supplementary Figure 4.

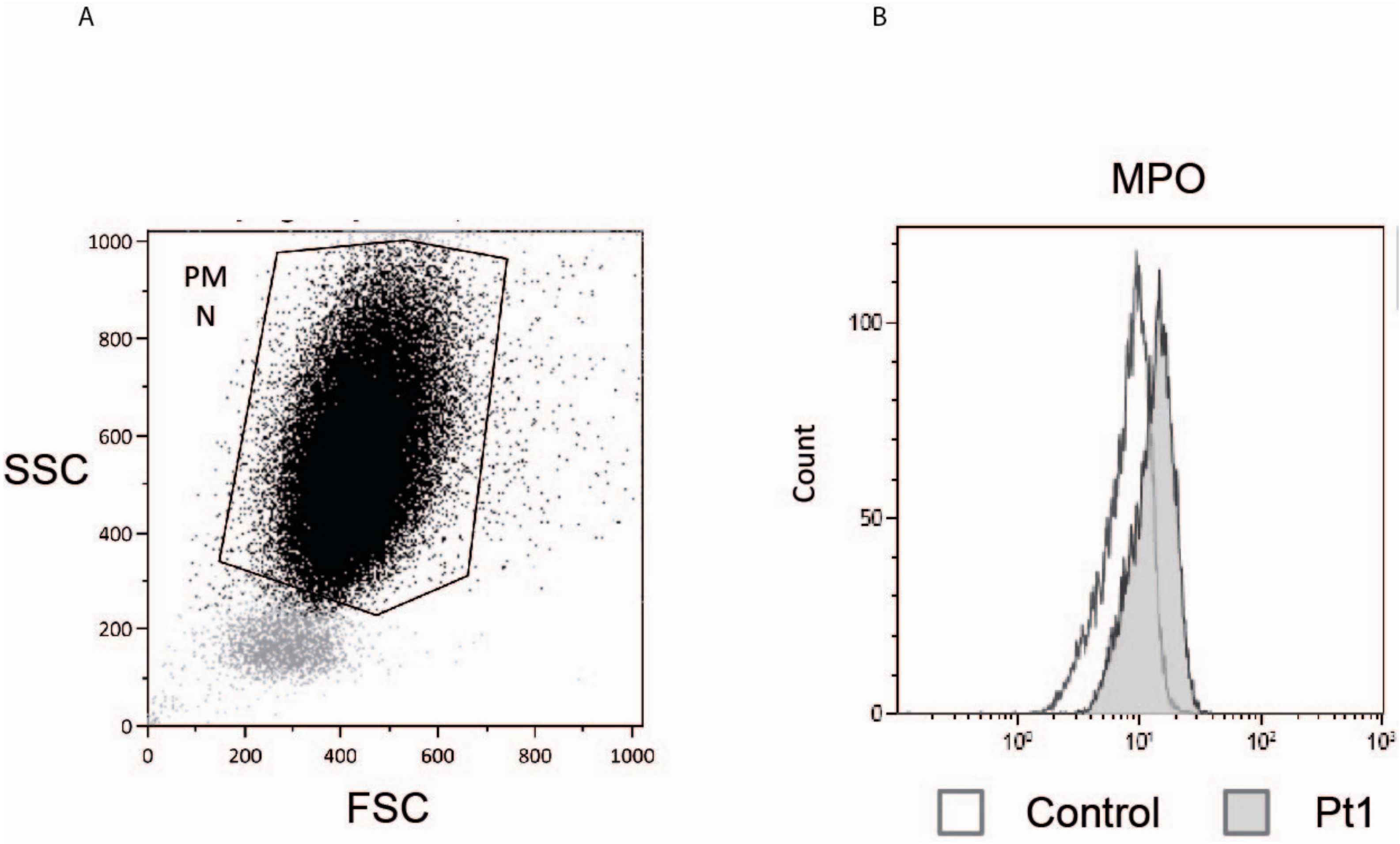

Myeloperoxidase (MPO) assessment. A. Scatter plot of whole blood cells, gated on polymorphonuclear cells (PMN). B. Histogram showing MPO intracellular expression in gated cells of control and ADA2 deficient patient 1.

Supplementary Figure 5.

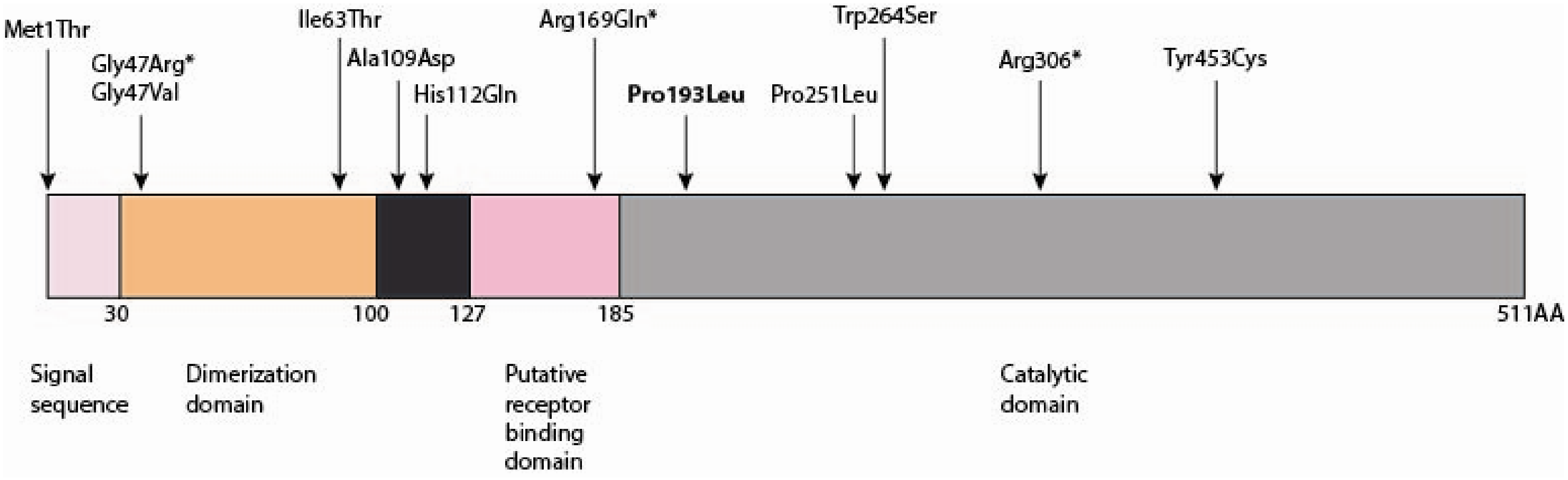

Schematic representation of the ADA2 protein showing the domain architecture and published pathological variants[1-3]. The new variant is shown in bold. Previously reported variants are marked by an asterix.

1. Zhou Q, Yang D, Ombrello AK, Zavialov AV, Toro C, Stone DL, Chae JJ, Rosenzweig SD, Bishop K, Barron KS, et al: **Early-onset stroke and vasculopathy associated with mutations in ADA2.** *N Engl J Med* 2014, **370:**911-920.
2. Navon Elkan P, Pierce SB, Segel R, Walsh T, Barash J, Padeh S, Zlotogorski A, Berkun Y, Press JJ, Mukamel M, et al: **Mutant adenosine deaminase 2 in a polyarteritis nodosa vasculopathy.** *N Engl J Med* 2014, **370:**921-931.
3. Garg N, Kasapcopur O, Foster J, 2nd, Barut K, Tekin A, Kizilkilic O, Tekin M: **Novel adenosine deaminase 2 mutations in a child with a fatal vasculopathy.** *Eur J Pediatr* 2014, **173:**827-830.
